# Cofactor-Activated Phosphorylation Is Required for Inhibition of Cortical Neuron Differentiation by Groucho/TLE1

**DOI:** 10.1371/journal.pone.0008107

**Published:** 2009-12-01

**Authors:** Manuel Buscarlet, Robert Hermann, Rita Lo, Yeman Tang, Kerline Joachim, Stefano Stifani

**Affiliations:** Centre for Neuronal Survival, Montreal Neurological Institute, McGill University, Montreal, Canada; Universidade Federal do Rio de Janeiro (UFRJ), Instituto de Biofísica da UFRJ, Brazil

## Abstract

**Background:**

Transcriptional co-repressors of the Groucho/transducin-like Enhancer of split (Gro/TLE) family regulate the expression of a variety of genes and are involved in numerous developmental processes in both invertebrate and vertebrate species. More specifically, Gro/TLE1 participates in mechanisms that inhibit/delay the differentiation of cerebral cortex neural progenitor cells into neurons during mammalian forebrain development. The anti-neurogenic function of Gro/TLE1 depends on the formation of protein complexes with specific DNA-binding transcription factors that engage Gro/TLE1 through WRP(W/Y) sequences. Interaction with those transcription partners results in Gro/TLE1 recruitment to selected DNA sites and causes increased Gro/TLE1 phosphorylation. The physiological significance of the latter event, termed “cofactor-activated phosphorylation,” had not been determined. Therefore, this study aimed at clarifying the role of cofactor-activated phosphorylation in the anti-neurogenic function of Gro/TLE1.

**Methods and Principal Findings:**

A combination of site-directed mutagenesis, mass spectrometry, biochemistry, primary cell culture, and immunocytochemical assays was utilized to characterize point mutations of Ser-286, a residue that is phosphorylated *in vivo* and is located within the serine/proline-rich (SP) domain of Gro/TLE1. Mutation of Ser-286 to alanine or glutamic acid does not perturb the interaction of Gro/TLE1 with DNA-binding partners, including the basic helix-loop-helix transcription factor Hes1, a prototypical anti-neurogenic WRP(W/Y) motif protein. Ser-286 mutations do not prevent the recruitment of Gro/TLE1 to DNA, but they impair cofactor-activated phosphorylation and weaken the interaction of Gro/TLE1 with chromatin. These effects are correlated with an impairment of the anti-neurogenic activity of Gro/TLE1. Similar results were obtained when mutations of Ser-289 and Ser-298, which are also located within the SP domain of Gro/TLE1, were analyzed.

**Conclusion:**

Based on the positive correlation between Gro/TLE1 cofactor-activated phosphorylation and ability to inhibit cortical neuron differentiation, we propose that hyperphosphorylation induced by cofactor binding plays a positive role in the regulation of Gro/TLE1 anti-neurogenic activity.

## Introduction

Groucho/transducin-like Enhancer of split (Gro/TLE) proteins are non-DNA binding transcriptional co-repressors that are recruited to gene regulatory sequences via interaction with a number of DNA-binding proteins. Together with specific partners, Gro/TLE family members mediate the gene regulatory functions of a variety of signalling pathways, including Notch, Wnt/Wingless, Transforming Growth Factor-β superfamily, and Epidermal Growth Factor receptor signal transduction mechanisms. As a result, invertebrate and vertebrate Gro/TLE proteins regulate a variety of developmental mechanisms and play important roles in integrating different signalling cascades [1]–[4].

A number of previous investigations have shown that Gro/TLE proteins are expressed in proliferating neural progenitor cells where they promote maintenance of the undifferentiated state by inhibiting/delaying neuronal differentiation [1], [2]. In *Drosophila melanogaster*, *gro* loss-of-function mutations cause the differentiation of supernumerary central and peripheral neurons [5]–[7]. This phenotype results from the disruption of the Notch-mediated lateral inhibition mechanism that normally restricts the number of neuroblasts within clusters of initially equipotential presumptive neural progenitor cells [8], [9]. Committed neuroblasts activate the Notch signalling pathway in adjacent cells, causing the transcriptional induction of genes encoding basic helix loop helix (bHLH) proteins of the Hairy/Enhancer of split (Hes) family. Hes proteins are DNA-binding factors that recruit Gro to repress the expression, as well as biochemical function, of pro-neuronal proteins encoded by the *achaete*-*scute* complex or *atonal* genes [8]–[11].

Similar mechanisms occur during mammalian neurogenesis. Gro/TLE proteins are expressed in proliferating neural progenitor cells in the developing murine central nervous system [12]–[15] and form complexes with mammalian Hes proteins [16], [17]. Transgenic mice with deregulated Gro/TLE1 expression exhibit an inhibition/delay of forebrain neuronal differentiation during embryonic development [18]. Moreover, forced Gro/TLE1 expression in undifferentiated cerebral cortex (cortical) neural progenitor cell cultures causes decreased neuronal differentiation and increased numbers of proliferating neural progenitors [19], [20].

The molecular mechanisms underlying the anti-neurogenic function of Gro/TLE1 in the developing mammalian forebrain are starting to be characterized. Previous work has shown that the ability of Gro/TLE1 to inhibit cortical neuron differentiation from undifferentiated stem/progenitor cells requires the capacity to interact with a particular group of transcription factors that bind to the Gro/TLE C-terminal WD40 repeat (WD) domain. These essential anti-neurogenic cofactors share the feature of recruiting Gro/TLE through short tetrapeptides typified by the sequence WRP(W/Y) [20]. Members of the WRP(W/Y) motif protein family include, but are not limited to, factors like Hes1, Hes3, and Hes5, which play essential roles in neural stem/progenitor cell maintenance and inhibition of neuronal differentiation [21]–[25].

The interaction of Gro/TLE1 with Hes1, as well as other transcription factors harbouring WRP(W/Y) motifs, has at least two consequences. It results in Gro/TLE1 recruitment to specific DNA sites [17], [19], [26] and induces Gro/TLE1 hyperphosphorylation [19], [27]. The latter effect, termed “cofactor-activated phosphorylation” [27], was also observed with other Gro/TLE family members [28]. The mechanisms underlying cofactor-activated phosphorylation of Gro/TLE proteins, as well as the biological role of this process, were not defined. Previous work has shown that cofactor-activated phosphorylation of Gro/TLE1 is blocked by deletion of the serine/proline-rich (SP) domain [19]. This domain contains many serine and threonine residues, several of which could be the substrate of phosphorylation. A role for the SP domain in Gro/TLE phosphorylation is in agreement with studies in *Drosophila* showing that Gro is phosphorylated within the SP domain at Ser-285 (equivalent to Ser-286 of Gro/TLE1) and Ser-297 (orthologous to Ser-298 of Gro/TLE1) [29]. However, the possible involvement of those SP domain serine residues in Gro/TLE cofactor-activated phosphorylation as well as the role of the latter modification in Gro/TLE activity remain to be defined.

Here we describe studies aimed at determining whether the SP domain of Gro/TLE1 is important for cofactor-activated phosphorylation and whether or not the latter has a positive or negative effect on Gro/TLE1 anti-neurogenic activity. Our results show that Ser-286 within the SP domain of Gro/TLE1 is phosphorylated *in vivo*. Mutation of Ser-286 into alanine or glutamic acid does not impair Gro/TLE1 ability to interact with anti-neurogenic WRP(W/Y) proteins like Hes1 and become recruited to DNA. However, these mutations abolish Gro/TLE1 cofactor-activated phosphorylation, weaken the association of Gro/TLE1 with chromatin, and block its anti-neurogenic activity. Similar effects were observed after mutation of SP domain Ser-289 and Ser-298. Taken together, these findings implicate Ser-286, Ser-289, and Ser-298 in the process of cofactor-activated phosphorylation. They suggest further that this post-translational modification plays a positive role in regulating the ability of Gro/TLE1 to inhibit neuronal differentiation.

## Methods

### Site Directed Mutagenesis and DNA Plasmids

DNAs encoding mutated forms of Gro/TLE1 harbouring the mutations S286A, S286E, S289A, S289E, S298A, and S298E were generated by site directed mutagenesis using the Quick Change II site directed mutagenesis kit (Stratagene, La Jolla, CA), using the *pCMV2-FLAG-Gro/TLE1* plasmid [19] as substrate. The following oligonucleotide primers were used for mutagenesis (mutations are underlined): S286A-F [5′- CTAAAGAAGGATGCTTCTAGCGCTCCAGCTTCCACGGCCTCCTC], S289A-F [5′ -CTAGCAGTCCAGCTGCCACGGCCTCCTC], S298A-F [5′-CCTCGGCAAGTTCCACTGCCTTGAAATCCAAAGAAATGAGC], S286E-F [5′-AGGATGCTTCTAGCGAACCAGCTTCCACGGCCTC], S289E-F [5′- CTTCTAGCAGTCCAGCTGAAACGGCCTCCTCGGCAAG] and S298E-F [5′-CCTCGGCAAGTTCCACTGAATTGAAATCCAAAGAAATGAGC]. *pcDNA3-GAL4dbd-Gro/TLE1* plasmids used for transcription assays were obtained by PCR amplification of the entire coding sequence of each mutant using the appropriate *pCMV2-FLAG-Gro/TLE1* plasmids as templates. This was followed by subcloning into the EcoRV site of *pcDNA3-GAL4dbd* plasmid, which encodes the DNA-binding domain of GAL4 (GAL4dbd). Vectors *pCMV2-FLAG-Hes1*, *pRc/CMV-Hes1*, *pCMV2-HA*-*Hes1*, *pCMV2-FLAG-Gro/TLE1*, *pCMV2-FLAG-Runx1*, *pCMV2-HA-Engrailed1 (En1)*, *pEGFP*, *p5xGAL4UAS-SV40promoter-luciferase*, *p6N-βy Ù-actinpromoter-luciferase*, *pFOX-Ngn3promoter-luciferase*, and *pRSV-β-galactosidase* were described [16], [17], [19], [20].

### Mass Spectrometry

Human embryonic kidney (HEK) 293 cells were transfected with *pCMV2-FLAG-Gro/TLE1* using the SuperFect reagent (Qiagen, Missisauga, ON, Canada) as described [17], [19]. FLAG-Gro/TLE1 was immunoprecipitated using an anti-FLAG antibody (Sigma, St. Louis, MI), followed by SDS-polyacrylamide gel electrophoresis and staining with Coumassie Blue. Resolved Gro/TLE1 bands were excised (1-mm cubes) and subjected to liquid chromatography-tandem mass spectrometry (LC-MS/MS) analysis at the McGill University/Genome Quebec Innovation Centre. Individual gel bands were washed twice with water, destained with 50% methanol in 100 mM ammonium bicarbonate, and then dehydrated with 75 µl acetonitrile. Samples were treated with 50 µl of 10 mM DTT for 30 minutes (60°C) followed by incubation with 50 µl of 55 mM iodoacetamide for 20 minutes (20°C). After washing and dehydration in ammonium bicarbonate and acetonitrile respectively, gel pieces were digested for 4.5 hours with 6 ng/µl of trypsin (Promega, Madison, WI) in 100 mM ammonium bicarbonate (pH 8.0). Peptides were extracted with 30 µl formic acid solution (1% formic acid in 2% acetonitrile) for 30 minutes. Peptides were extracted twice more with 12 µl formic acid solution and 12 µl acetonitrile for 30 min for a final volume of 60 ml.

The resulting peptides (20 µl) were loaded onto Zorbax 300SB-C18 5×0.3 mm desalting columns (Agilent, Mississauga, Ontario) and washed for 5 min at 15 µl/min with 3% acetonitile: 0.1% formic acid. Peptide separation was performed on a New Objectives Biobasic C18 10×0.075 mm Integrafrit analytical column (New Objectives, Woburn, MA). Gradient elution was from 10% acetonitrile: 0.1% formic acid to 95% acetonitrile: 0.1% formic acid in 30 min using an Agilent 1100 Nanoflow system. The chromatographic effluent was introduced at a flow rate of 200 nL/min.

MS was performed using a QTRAP 4000 instrument from Sciex-Applied Biosystems (Foster City, CA). The QTRAP 4000 system was operated in positive ion mode and spectra acquired in a data-dependent manner, with the top three most intense ions in the MS survey scan selected for MS/MS by collision-induced dissociation. Precursor ions selected two times were excluded for 90 seconds. Survey scan used was enhanced MS scan from 375 to 1600 m/z at 4000 amu/sec using Dynamic Fill time. Collision energy was determined using a rolling collision energy equation. MS/MS data were acquired for three scans from 70 to 1700 m/z with 20 ms trap fill time and Q0 trapping activated.

Peak lists were generated with mascot distiller 2.1 and searched against the IPI human database (67770 sequences) from September 2007. Mascot 2.1 search parameters used were trypsin with a single miscleavage, carboxyamidomethylation of cysteines as fixed modification, oxydation of methionines and phosphorylation of serine, threonine and tyrosine as variable modifications, 1.5 Da precursor and 0.8 MS/MS fragment tolerances.

### Co-Immunoprecipitation Assays and Phosphatase Treatment

HEK293 cells were co-transfected with 1.0 µg of *pCMV2-FLAG-Gro/TLE1 (WT, S286A*, *S286E*, *S289A*, *S289E*, *S298A*, *S298E)* and 1.0 µg of either *pCMV2-FLAG-HES1* or *pCMV2-HA-En1*. Cell lysates were prepared and co-immunoprecipitations using either anti-HA (Covance, Berkeley, CA) or anti-Gro/TLE1 [19] antibodies were performed as described [19], [20], [30]. This step was followed by Western blotting analysis using anti-FLAG (1∶10,000) or anti-HA (1∶5,000) antibodies. Incubation of cell extracts with calf intestinal phosphatase was as described previously [31].

### Transient Transfection/Transcription Assays

HEK293 cells were transfected using the SuperFect reagent. In all cases, the total amount of transfected DNA was adjusted at 3.0 µg per well using *pcDNA3*. In studies using a GAL4 responsive promoter, assays were performed with 1.5 µg/transfection of reporter construct *p5xGAL4UAS-SV40promoter-luciferase* in the presence or absence of plasmids *pcDNA3-GAL4dbd* or *pcDNA3-GAL4dbd-Gro/TLE1* (wild-type or mutated sequences) (1.0 µg/transfection). Assays using the Hes1-regulated reporter vectors *p6N-β-actinpromoter-luciferase* [17] and *pFOX-Ngn3promoter-luciferase* [32], [33] (2.0 µg/transfection), were performed with pCMV2-FLAG-Hes1 (0.050 µg/transfection) in the absence or presence of *pCMV2-FLAG-Gro/TLE1* (wild-type or mutated sequences) (0.1 µg/transfection). In each case, 0.25 µg/transfection of β-galactosidase expression plasmid, *pRSV-βgal*, was used to normalize for transfection efficiency. Twenty-four hours after transfection, cells were subjected to determination of luciferase activity as described [17], [19], [32]. Results were expressed as mean values±S.D. Expression of GAL4dbd-Gro/TLE1 fusion proteins was detected using an anti-GAL4dbd antibody (1∶500; Santa Cruz Biotechnology). Expression of FLAG epitope-tagged proteins was detected with an anti-FLAG antibody.

### Preparation of Subcellular Fractions

Postnuclear supernatants and chromatin-enriched fractions were obtained as described previously [27], [31]. Subcellular fractions were analyzed by Western blotting using anti-FLAG, anti-Gro/TLE (‘pan-TLE’) [19], [27], [31], anti-p65 (1∶3,000; Santa Cruz Biotechnology, Santa Cruz, CA), and anti-histone deacetylase 1 (HDAC1) (1∶1,000; Santa Cruz Biotechnology) antibodies. Experiments using the protein kinase CK2 inhibitor, chrysin (Sigma) were performed as described [27].

### Chromatin Immunoprecipitation

Chromatin immunoprecipitation experiments were performed as described in Supplemental Methods S1.

### Cortical Neural Progenitor Cell Cultures

Animal studies followed the guidelines of the Canadian Council of Animal Care and were approved by the Montreal Neurological Institute Animal Care Committee. Primary cultures of neural progenitor cells from embryonic day (E) 12–14 mouse dorsal telencephalic cortices were obtained and cultured exactly as described [19], [20], [33]. Cells were transfected after 48 hours in vitro as described [19], [20] using plasmids encoding either enhanced green fluorescent protein (GFP) alone (0.2 µg/well), or both GFP (0.2 µg/well) and Gro/TLE1 [*pCMV2-FLAG-Gro/TLE1 (WT, S286A, S289A, S298A, S286E*, *S289E*, or *S298E*)] (0.8 µg/well). Three days after transfection, cells were analyzed by immunocytochemistry using antibodies against the proliferating cell marker Ki67 (1∶200; BD Pharmigen), the neural progenitor cell marker nestin (1∶400; Millipore), the neuronal cell marker type III β-tubulin (1∶300; Promega, Madison, WI), and the neuronal cell marker neuron specific nuclear protein (NeuN) (1∶100; Millipore). Cells were counterstained with Hoechst 33258 (Sigma) before examination by fluorescence microscopy [19], [20], [33]. Grayscale images were digitally assigned to the appropriate red or green channel using Northern Eclipse software (Empix, Missisauga, ON, Canada). Three to six random fields of each condition (per experiment) were used for quantitation of the percent of GFP-positive cells co-expressing specific markers [32]–[36]. Results were expressed as the mean values±the standard deviation. At least six separate experiments were conducted in each case and statistical analysis was performed using the Student's *t*-Test.

## Results

### Phosphorylation of the N-Terminal Portion of the Gro/TLE1 SP Domain *In Vivo*

**Figure 1:**
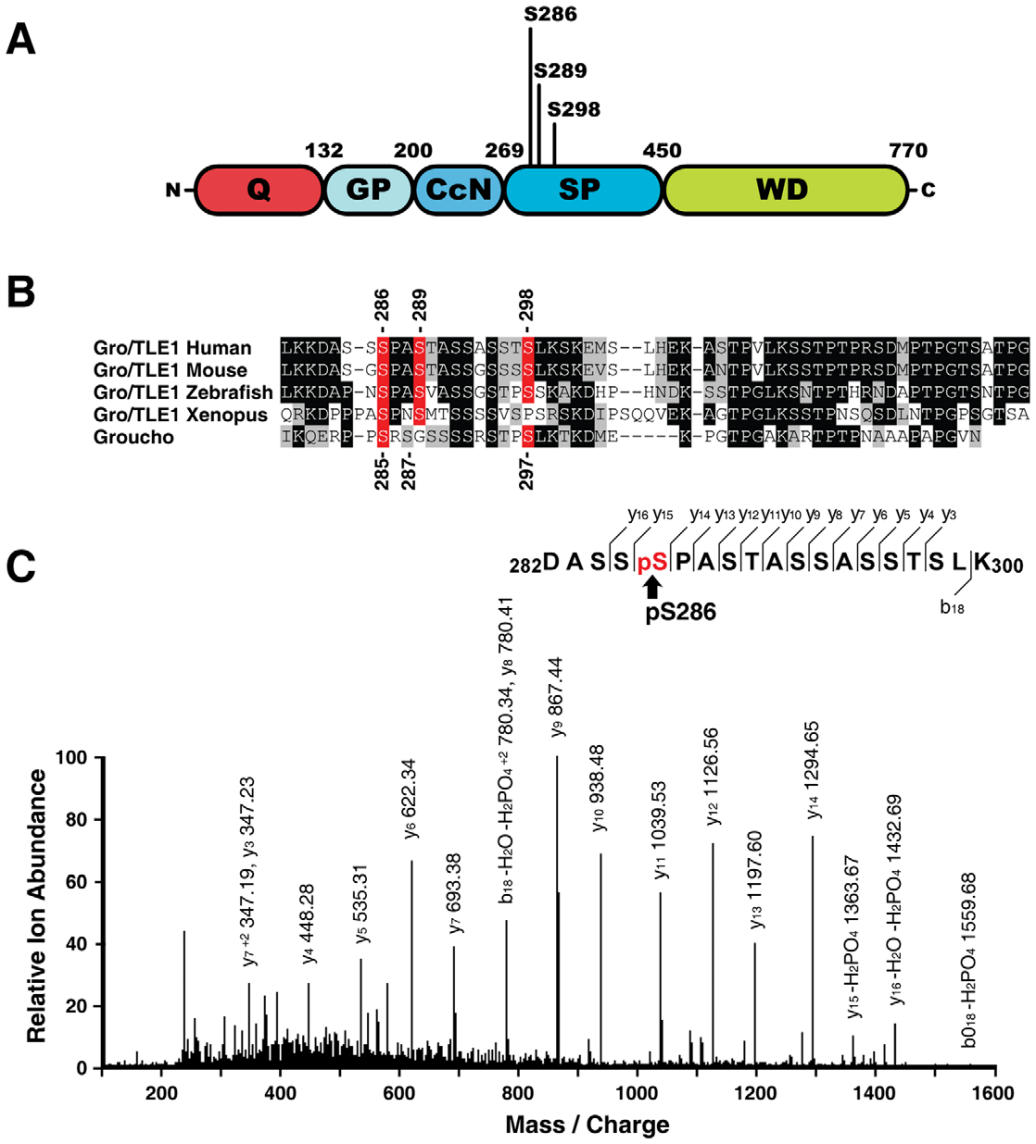
Phosphorylation of Gro/TLE1 SP domain. (A) Schematic representation of the Gro/TLE1 domain structure characterized by glutamine-rich (Q), glycine/proline-rich (GP), protein kinase CK2/cell cycle-dependent kinase 2/nuclear localization sequence (CcN), serine/proline-rich (SP), and WD40 repeat (WD) domains. (B) Sequence comparison of the N-terminal portion of the SP domain of human, mouse, zebrafish, and *Xenopus* Gro/TLE1, as well as *Drosophila* Gro. The location of Ser-286, Ser-289, and Ser-298 within the SP domain of Gro/TLE1 is indicated (top), as is the location of Ser-285, Ser-287, and Ser-298 of *Drosophila* Gro (bottom). Identical and conserved residues are highlighted in black or grey, respectively. (C) Tandem mass spectrum of the indicated phosphopetide from human Gro/TLE1; a sufficient number of Y_n_ ions (C-terminus-derived fragment ions) were detected to assign the phosphorylation site to Ser-286.

Gro/TLE1 is a basally phosphorylated transcription factor that undergoes increased phosphorylation upon interaction with a variety of DNA-binding proteins. This hyperphosphorylation event, termed cofactor-activated phosphorylation [19], [27], is blocked by deletion of amino acids 285–335 [19]. Those residues are part of the N-terminal portion of the Gro/TLE1 SP domain (Fig. 1A) and define a region with a high density of possible phosphorylation sites, several of which are evolutionarily conserved from flies to humans (Fig. 1B). To determine whether amino acids 285–335 define a phosphorylation domain, we expressed FLAG epitope-tagged Gro/TLE1 in HEK293 cells. Immunoprecipitated Gro/TLE1 was digested with trypsin and subjected to LC-MS/MS mass spectrometry. Among those tryptic peptides that were amenable to analysis, searching against the IPI database led to the identification of a tryptic phosphopeptide (^282^DASSpSPASTASSASSTSLK^300^) that was derived from the SP domain of Gro/TLE1 and contained a phosphorylated serine at position 286 (Fig. 1C). Ser-286 of Gro/TLE1 is evolutionarily conserved (Fig. 1B) and corresponds to *Drosophila* Gro Ser-285, a residue that was identified as a site of phosphorylation by two-dimensional phosphopeptide analysis [29]. We detected phosphorylation of Ser-286 of Gro/TLE1 even without co-transfection of Gro/TLE-binding proteins such as Hes1 (data not shown). This situation suggests that phosphorylation of Ser-286 is not dependent on cofactor binding, although it should be emphasized that HEK293 cells endogenously express a number of Gro/TLE-binding proteins, including Hes1 [36]. Our analysis failed to detect peptides phosphorylated at Ser-289 and/or Ser-298, which were hypothesized to be sites of phosphorylation based on the analysis of *Drosophila* Gro [29]. It is possible that hyperphosphorylated peptides were too hydrophilic to be retained on reversed-phase columns. Together, these observations show that the SP domain of Gro/TLE1 is phosphorylated at position 286.

### Characterization of Point Mutations of Serine Residues in the SP Domain of Gro/TLE1

**Figure 2:**
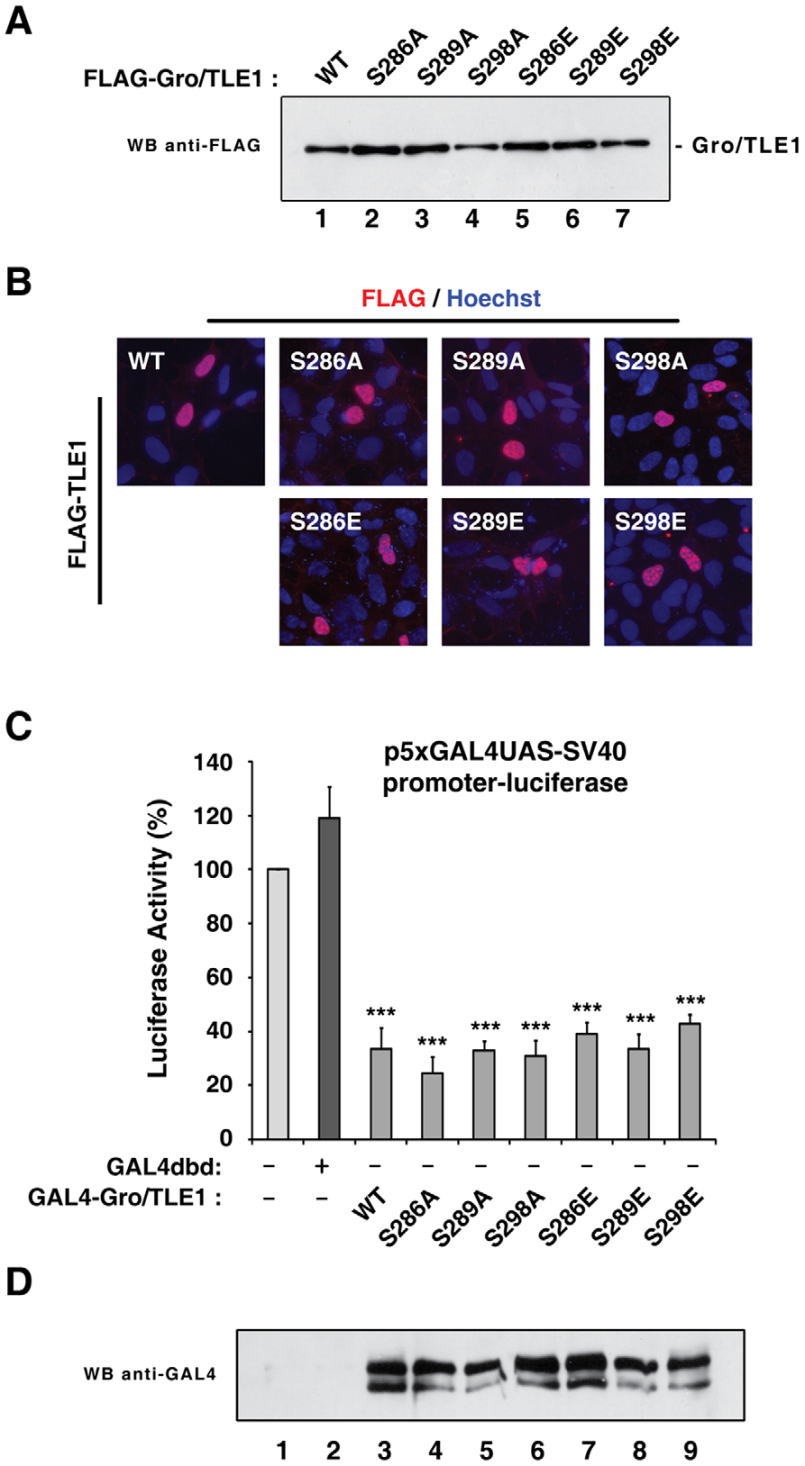
Characterization of Gro/TLE1 SP domain mutations. (A) Wild type (WT) or mutated forms of FLAG epitope-tagged Gro/TLE1 were expressed in HEK293 cells, followed by cell lysis and fractionation on a 10% SDS polyacrylamide gel and Western blotting (WB) analysis using an anti-FLAG antibody. (B) Wild type or mutated Gro/TLE1 proteins were expressed in HEK293 cells followed by immunofluorescence analysis using an anti-FLAG antibody. (C) HEK293 cells were transfected with a *p5xGAL4UAS-SV40promoter-luciferase* reporter construct (1.5 µg/transfection) in the absence (*bars 1 and 2*) or presence of wild type (*bar 3*) or mutated (*bars 4–9*) forms of GAL4dbd-Gro/TLE1 (1 µg/transfection). Luciferase activity in the absence of effector plasmids was considered 100% and values in the presence of effector plasmids were expressed as the mean±the standard deviation of at least four separate experiments performed in duplicate; (***, *P*<0.0001). (D) Western blotting analysis of GAL4dbd-Gro/TLE1 proteins used in the transcription assays using an anti-GAL4dbd antibody; GAL4dbd-Gro/TLE1 proteins migrate as a doublet, as shown previously (20).

Based on the mass spectrometric analysis of Gro/TLE1, we selected Ser-286 as an *in vitro* mutagenesis target. Moreover, because of previous studies in *Drosophila* suggesting that Gro is phosphorylated at Ser-287 (analogous to Gro/TLE1 Ser-289) and Ser-297 (orthologous to Gro/TLE1 Ser-298) [29] (Fig. 1B), we selected Ser-289 and Ser-298 in the SP domain of Gro/TLE1 as additional mutagenesis targets. Each serine residue was replaced by either alanine or glutamic acid (these mutations will be hereafter collectively termed “SP domain mutations”). All mutated proteins displayed normal electrophoretic mobility on denaturing polyacrylamide gels, with some occasional variation in expression levels (Fig. 2A). Moreover, they all translocated to the nucleus like wild type Gro/TLE1 (Fig. 2B). More importantly, the mutated proteins retained the ability to repress transcription from a basally active promoter when expressed as fusion proteins with the GAL4 DNA-binding domain (Fig. 2C and D). Together, these results show that mutation of Ser-286, Ser-289, and Ser-298 does not detectably perturb the folding and biochemical activity of Gro/TLE1.

### SP Domain Mutations Do Not Perturb the Ability of Gro/TLE1 to Interact with WRPW or Eh1 Repressor Peptides

**Figure 3:**
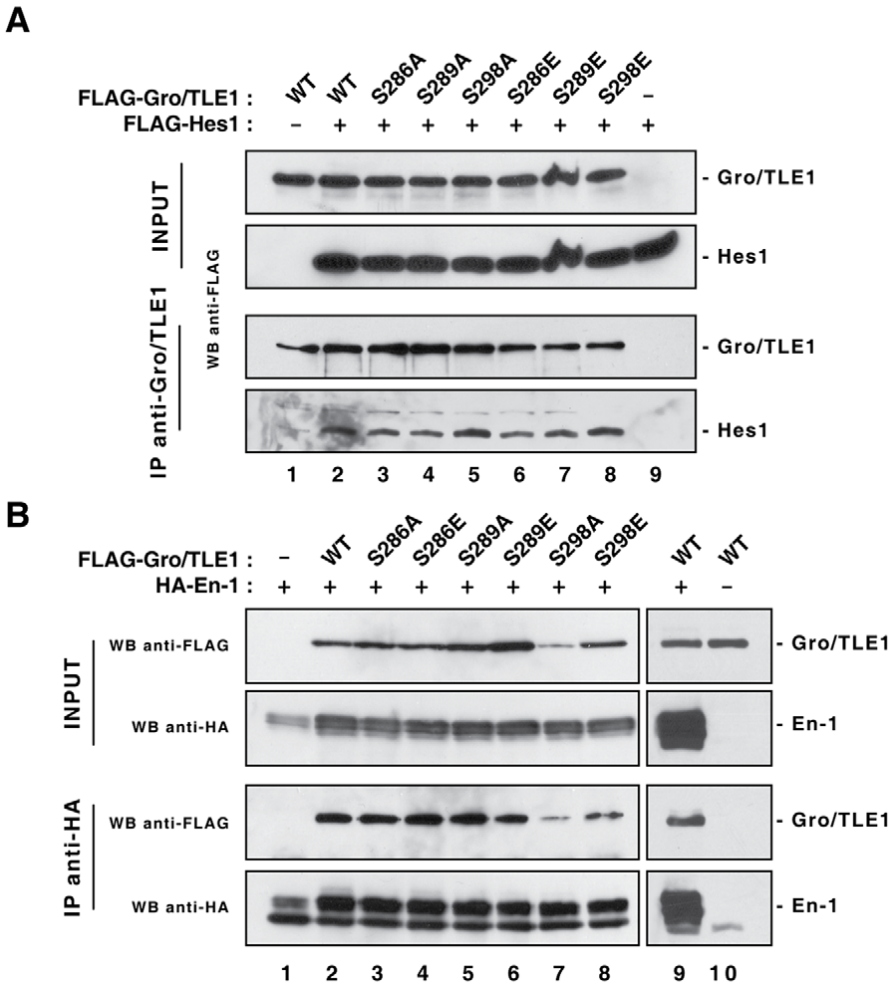
Effect of SP domain mutations on Gro/TLE1 interaction with Hes1 and Engrailed1. FLAG epitope-tagged wild type (WT) or mutated Gro/TLE1 proteins were co-expressed in HEK293 cells with FLAG-Hes1 (A) or HA epitope-tagged En1 (B), as indicated. Each cell lysate (INPUT) was subjected to immunoprecipitation (IP) with either anti-Gro/TLE1 (A) or anti-HA (B) antibodies. Immunoprecipitates, together with 1/10 of each input lysate, were fractionated on a 10% SDS polyacrylamide gel, followed by Western blotting (WB) with the indicated antibodies.

Gro/TLE proteins interact with a variety of transcription factors that harbour sequences belonging to the WRP(W/Y) or Engrailed homology 1 (Eh1) repressor peptide families [1]. Both WRP(W/Y) and Eh1 peptides bind to the C-terminal WD domain of Gro/TLE [20], [37]. This situation suggested that mutation of residues 286, 289, or 298 should not affect binding of Gro/TLE1 to cofactors containing those repressor peptides. To examine this possibility, co-immunoprecipitation assays were performed using Hes1 as a prototypical example of WRP(W/Y) motif proteins. Interaction with WRP(W/Y) motif proteins was shown to be essential to the anti-neurogenic function of Gro/TLE1 [20]. En1 was used in similar assays as a typical example of Eh1 motif-bearing proteins. These experiments showed that all Gro/TLE1 SP domain mutations retained the ability to bind to Hes1 and En1 in co-immunoprecipitation assays (Fig. 3A and B).

**Figure 4:**
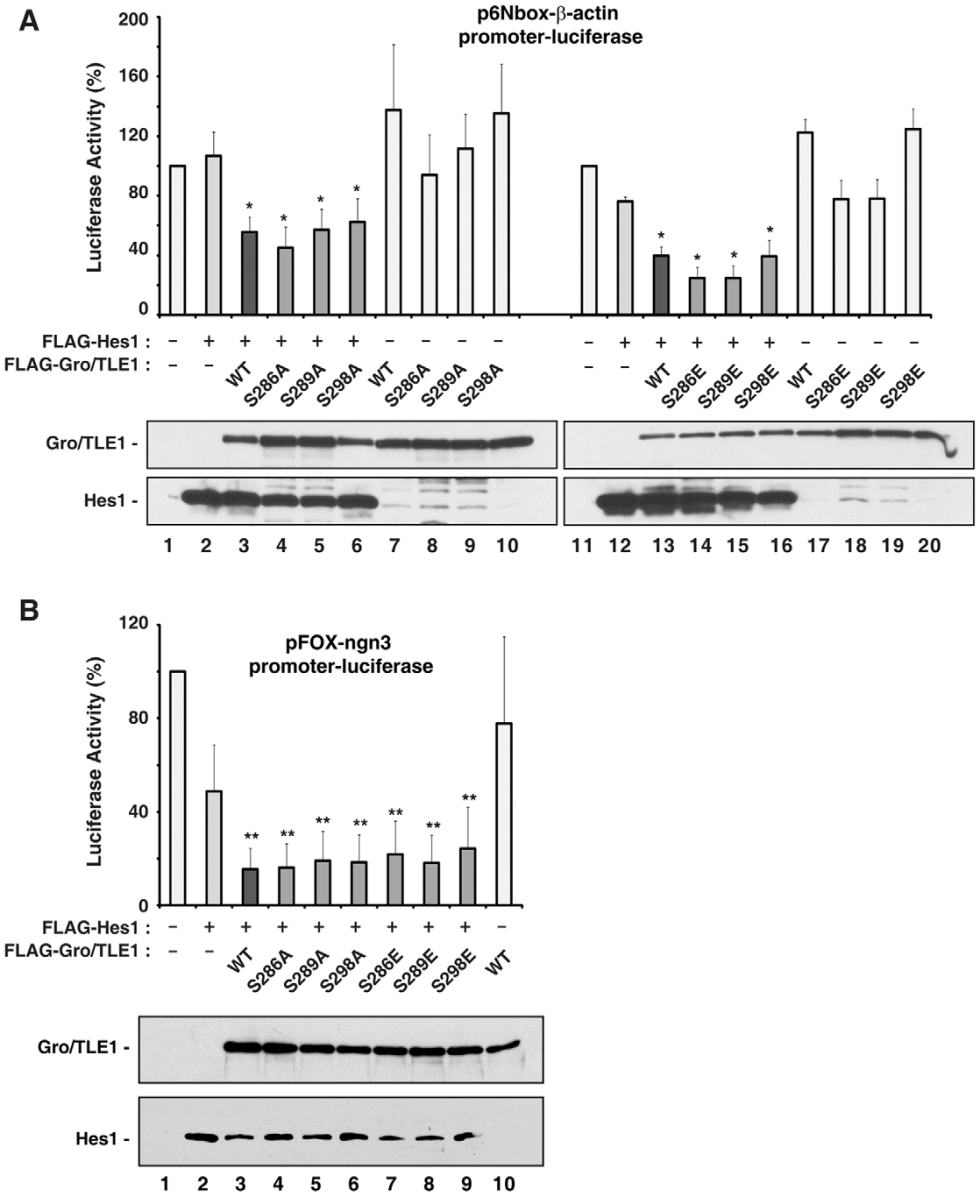
Effect of SP domain mutations on Gro/TLE1 transcriptional co-repressor activity in transiently transfected cells. HEK293 cells were transfected with reporter constructs containing the *luciferase* gene under the control of either the *β-actin* promoter linked to six tandem N-boxes (A) or a ∼3.0 kb fragment of the *neurogenin3* promoter, which contains multiple N-boxes (B). Reporter plasmids were transfected alone (*bar 1*; luciferase activity considered 100%) or in combination with the following expression plasmids: pCMV2-FLAG-Hes1 (50 ng/transfection), pCMV2-FLAG-Gro/TLE1 (WT, S286A, S289A, S298A, S286E, S289E, or S298E) (100 ng/transfection), as indicated. Luciferase activities were expressed as the mean±the standard deviation of at least four separate experiments performed in duplicate; (*, *P*<0.01; **, *P*<0.001). Western blotting analysis of Gro/TLE1 and Hes1 proteins tested in the transcription assays, using an anti-FLAG antibody, is shown under each graph.

To further test if the SP domain mutations would interfere with the ability of Gro/TLE1 to functionally interact with Hes1, transient transfection/transcription experiments were performed next to determine whether the mutated proteins were still competent to repress transcription together with Hes1. These experiments were performed recognizing that transient transfection/transcription assays are useful to monitor the ability of different transcription factors to regulate transcription together, but are of limited usefulness to study the regulation of endogenous genes because it is unlikely that transfected reporter plasmids acquire true chromatin-like structures. HEK293 cells were transfected with two separate reporter constructs containing the *luciferase* gene under the control of either a basally active *β-actin* promoter linked to six tandem Hes1-binding sites (N-box) (Fig. 4A) or a 3.0 kb fragment of the *neurogenin3* promoter containing multiple N-box sequences (Fig. 4B). Hes1 was shown to repress transcription from both of those promoters in a WRPW motif-dependent manner [17], [32]. We designed experimental conditions where little or no transcriptional repression was observed when Hes1 was expressed alone. Co-expression of wild-type Gro/TLE1 resulted in a significant increase in transcriptional repression in the presence of Hes1 (Fig. 4A, cf. bars 1–3 and 11–13; 4B, cf. bars 1–3). Gro/TLE1 had no effect on reporter gene transcription when expressed in the absence of Hes1 (Fig. 4A, bars 7 and 17; 4B, bar 10). Similar to wild type Gro/TLE1, each SP domain mutant tested in our assays was competent to promote Hes1-mediated repression (Fig. 4A and B). Taken together, these results show that the SP domain mutations do not disrupt the binding of Gro/TLE1 to Hes1.

### SP Domain Mutations Perturb Cofactor-Activated Phosphorylation of Gro/TLE1

**Figure 5:**
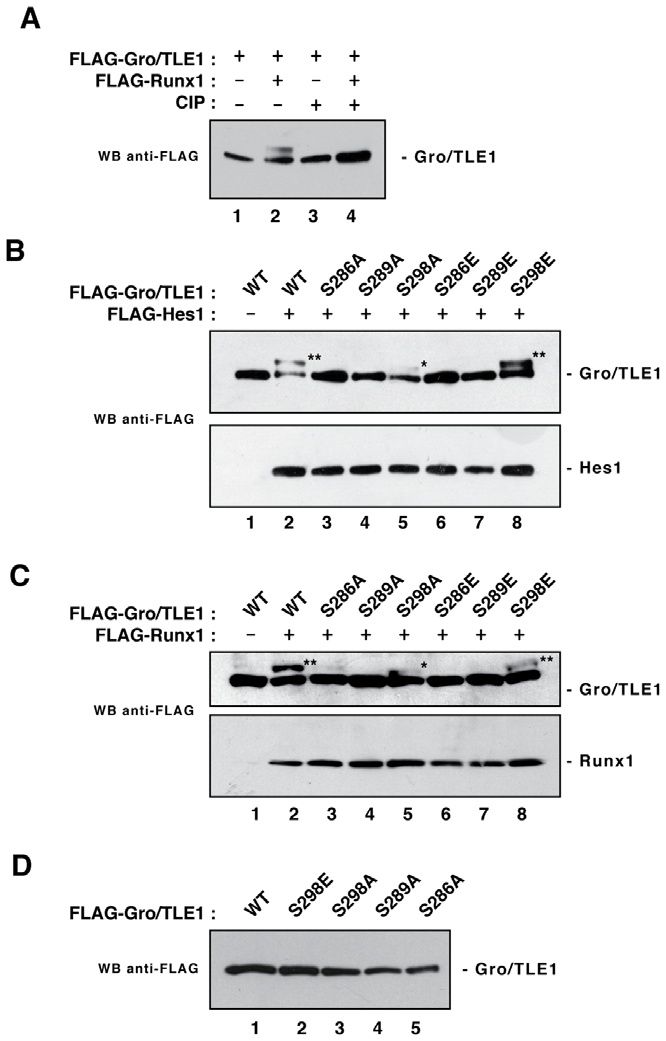
Effect of SP domain mutations on cofactor-activated phosphorylation of Gro/TLE1. (A) Equivalent amounts of protein extracts from cells transfected with the indicated proteins were incubated in the absence (*lanes 1* and *2*) or presence (*lanes 3* and *4*) of calf intestinal phosphatase (CIP), followed by fractionation on a 6% SDS polyacrylamide gel and Western blotting (WB) analysis with an anti-FLAG antibody. Phosphatase treatment abolished the slower Gro/TLE1 species induced by Runx1 expression. (B and C) HEK293 cells were co-transfected with FLAG epitope-tagged wild type or mutated Gro/TLE1 proteins in the absence or presence of either FLAG-Hes1 (B) or FLAG-Runx1 (C), followed by fractionation on a 6% SDS polyacrylamide gel and Western blotting with an anti-FLAG antibody. The slower Gro/TLE1 band visible when Hes1 or Runx1 were co-expressed (*lanes 2* and *8*; two asterisks) was either reduced (lane 5; one asterisk) or absent in the case of all SP domain mutations (*lanes 3–7*), except for S298E (*lane 8*). (D) Western blotting analysis with an anti-FLAG antibody of the indicated proteins expressed in HEK293 cells and fractionated on a 5% SDS polyacrylamide gel.

We next examined the effect of individual SP domain mutations on the ability of Gro/TLE1 to undergo cofactor-activated phosphorylation induced by proteins containing WRP(W/Y) repressor peptides (Fig. 5A). Mutation of Ser-286 and Ser-289, to either alanine or glutamic acid, blocked Gro/TLE1 hyperphosphorylation induced by Hes1 (harbouring a WRPW peptide) or Runx1 (WRPY peptide) (Fig. 5B and C, cf. lanes 2–4, 6 and 7). The effect of mutating Ser-298 was more complex. The S298A mutation did not abolish cofactor-activated phosphorylation, but the change in Gro/TLE1 electrophoretic mobility was consistently less pronounced than in the case of wild type Gro/TLE1 (Fig. 5B and C, cf. lanes 2 and 5). The S298E mutation did not perturb cofactor-activated phosphorylation, which was readily observed in the presence of both Hes1 and Runx1 (Fig. 5B and C, lane 8). Gro/TLE1(S298E) migrated like wild type Gro/TLE1 on low percentage SDS-polycrylamide gels when expressed in the absence of cofactor, showing no signs of constitutive hyperphosphorylation (Fig. 5D). The same results were obtained when other Gro/TLE1-binding proteins were tested (data not shown). Together, these findings show that both Ser-286 and Ser-289 are necessary for cofactor-activated phosphorylation of Gro/TLE1. Replacing these residues with glutamic acid is not sufficient to replace the lack of a serine residue. In contrast, a serine-glutamic acid substitution at position 298 does not affect cofactor-activated phosphorylation, suggesting that Ser-298 is not phosphorylated during that process but needs to be phosphorylated for cofactor-activated phosphorylation to occur.

### SP Domain Mutations Weaken the Binding, but Not the Recruitment, of Gro/TLE1 to Chromatin

**Figure 6:**
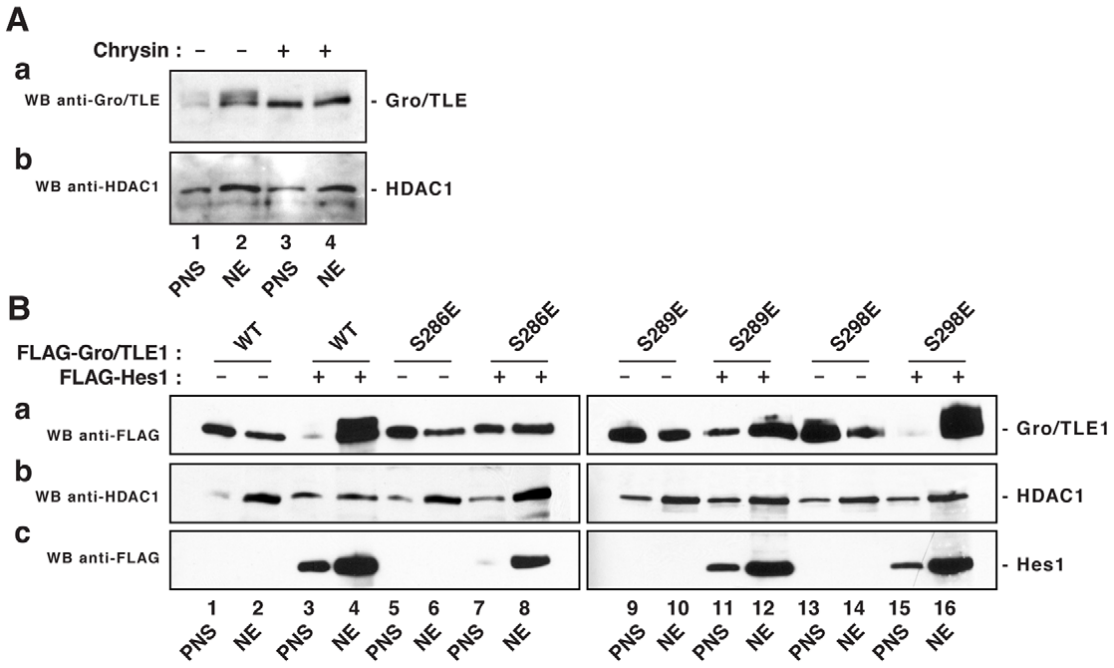
Effect of SP domain mutations on the association of Gro/TLE1 with chromatin. (A) Pharmacological inhibition of protein kinase CK2. A subcellular fractionation procedure was performed to obtain postnuclear supernatants (PNS) and chromatin-enriched nuclear extracts (NE) from cells transfected with an Hes1-expression plasmid to promote the cofactor activated hyperphosphorylation of endogenous Gro/TLE proteins. Equivalent amounts of fractions were subjected to SDS-polyacrylamide gel electrophoresis (6% gel), followed by sequential Western blotting (WB) with anti-Gro/TLE (‘panTLE’) (a) and anti-HDAC1 (b) antibodies. Treatment with chrysin resulted in reduced nuclear retention of endogenous Gro/TLE, but not HDAC1, proteins. It also caused a decrease of the more slowly migrating, hyperphosphorylated Gro/TLE form(s), as previously reported (27). (B) Analysis of the chromatin association of Gro/TLE1 by subcellular fractionation. Postnuclear supernatant and chromatin-enriched nuclear extracts from cells transfected with the indicated combinations of FLAG-tagged proteins were subjected to SDS-polyacrylamide gel electrophoresis, followed by sequential Western blotting with anti-FLAG (a and c) and anti-HDAC1 (b) antibodies, as indicated. Both the hyperphosphorylation (readily detactable on 6% gels) and increased chromatin association of wild type Gro/TLE1 induced by Hes1 were impaired by the SP mutations S286E and S289E (cf. lanes 3 and 4 to lanes 7 and 8, 11 and 12), but not by the S298E mutation (cf. lanes 3 and 4 to lanes 15 and 16). No changes were observed in the strength of the nuclear association of Hes1 and HDAC1 (panels b and c). Hes1 was consistently expressed at slightly lower levels when co-expressed with the S286E mutant. Shown is a representative example of three separate experiments.

Gro/TLE proteins can translocate to the nucleus but have no intrinsic DNA-binding ability. They become recruited to chromatin by interacting with specific cofactors, such as Hes1. Previous studies have suggested that cofactor-activated phosphorylation promotes the association of Gro/TLE proteins with chromatin. More specifically, hyperphosphorylated Gro/TLEs are preferentially recovered in chromatin-enriched nuclear fractions after subcellular fractionation experiments, whereas underphosphorylated forms are found mostly in postnuclear supernatant fractions [27], [31], [38]. Moreover, cofactor-activated phosphorylation of Gro/TLE is dependent on a previous constitutive phosphorylation event mediated by protein kinase CK2 [27]. Pharmacological inhibition of protein kinase CK2 has two effects: it impairs the ability of Gro/TLE to undergo cofactor-activated phosphorylation and causes a weakened interaction of Gro/TLE with chromatin, as shown by comparing the ‘retention’ of Gro/TLE in chromatin-enriched nuclear fractions obtained after subcellular fractionation in the absence or presence of protein kinase CK2 inhibitors (Fig. 6A) [27].

Based on these observations, we examined whether the SP domain mutations that block cofactor-activated phosphorylation would weaken the strength of the interaction of Gro/TLE1 with chromatin. Subcellular fractionation assays were performed to compare the nuclear retention of wild type or mutated Gro/TLE1 proteins in the absence or presence of Hes1. All Gro/TLE1 proteins tested were poorly retained in the chromatin-enriched nuclear fraction in the absence of Hes1, resulting in the recovery of considerable amounts of proteins in the post-nuclear supernatant (Fig. 6Ba, cf. lanes 1 and 2, 5 and 6, 9 and 10, 13 and 14). The co-expression of Hes1 led to both cofactor-activated phosphorylation of Gro/TLE1 and the retention of hyperphosphorylated Gro/TLE1 in the chromatin fraction, indicative of a tighter association with chromatin components (Fig. 6Ba, cf. lanes 3 and 4). In contrast, significant amounts of the S286E mutant, which did not undergo cofactor-activated phosphorylation, were recovered in the postnuclear supernatant even in the presence of Hes1 (Fig. 6Ba, cf. lanes 7 and 8). Similar results were obtained when mutation S286A was tested (Figure S1a). Mutations S289A, S289E, and S298A also resulted in decreased nuclear retention of Gro/TLE1 (Fig. 6Ba and Figure S1a). These differences were specific because we observed no detectable changes in the subcellular localization of co-transfected Hes1 or endogenous proteins like histone deacetylase 1 or the NF-κB subunit p65 (Fig. 6B, b and c; Figure S1). In contrast, mutant S298E, which was competent to undergo cofactor-activated phosphorylation, was mostly retained in the chromatin fraction like wild type Gro/TLE1 (Fig. 6Ba, cf. lanes 15 and 16).

To exclude the possibility that the observed differences might be due to perturbations of the recruitment of Gro/TLE1 to DNA, we performed chromatin immunoprecipitation assays. These experiments involve a covalent cross-linking step that ‘immobilizes’ on chromatin the entire complex of Gro/TLE1 and its DNA-binding partners and other cofactors. As a consequence, the association of Gro/TLE1 with DNA depends only in part on the affinity of Gro/TLE1 for chromatin but mostly on the cross-linking of the entire transcription complex, including the DNA-binding factors to which Gro/TLE1 is bound. We found that wild type and SP domain mutated Gro/TLE1 proteins were all able to become localized *in vivo* to the promoter of the *Ascl1/Mash1 (Ascl1)* gene, a previously characterized [26] *bona fide* target of Hes1: Gro/TLE complexes (Figure S2). This finding is in agreement with the demonstration that Gro/TLE1 folding and ability to form complexes with Hes1 are not affected by these mutations. Taken together, these results provide evidence that mutations that impair cofactor-activated phosphorylation do not prevent the recruitment of Gro/TLE1 to physiologically relevant promoters, but weaken the strength of Gro/TLE1 interaction with chromatin components.

### Cofactor-Activated Phosphorylation Is Required for Gro/TLE1 Anti-Neurogenic Activity

Gro/TLE1 inhibits the differentiation of cortical neurons from undifferentiated progenitors [19], [20]. This function requires complex formation with proteins containing the WRP(W/Y) motif but is not dependent on interactions with Eh1 motif-bearing transcription factors [20]. The characterization of SP domain mutations that do not affect interaction with WRP(W/Y) proteins but block cofactor-activated phosphorylation of Gro/TLE1 provided a means of assessing the role of the latter mechanism in Gro/TLE1 anti-neurogenic activity.

**Figure 7:**
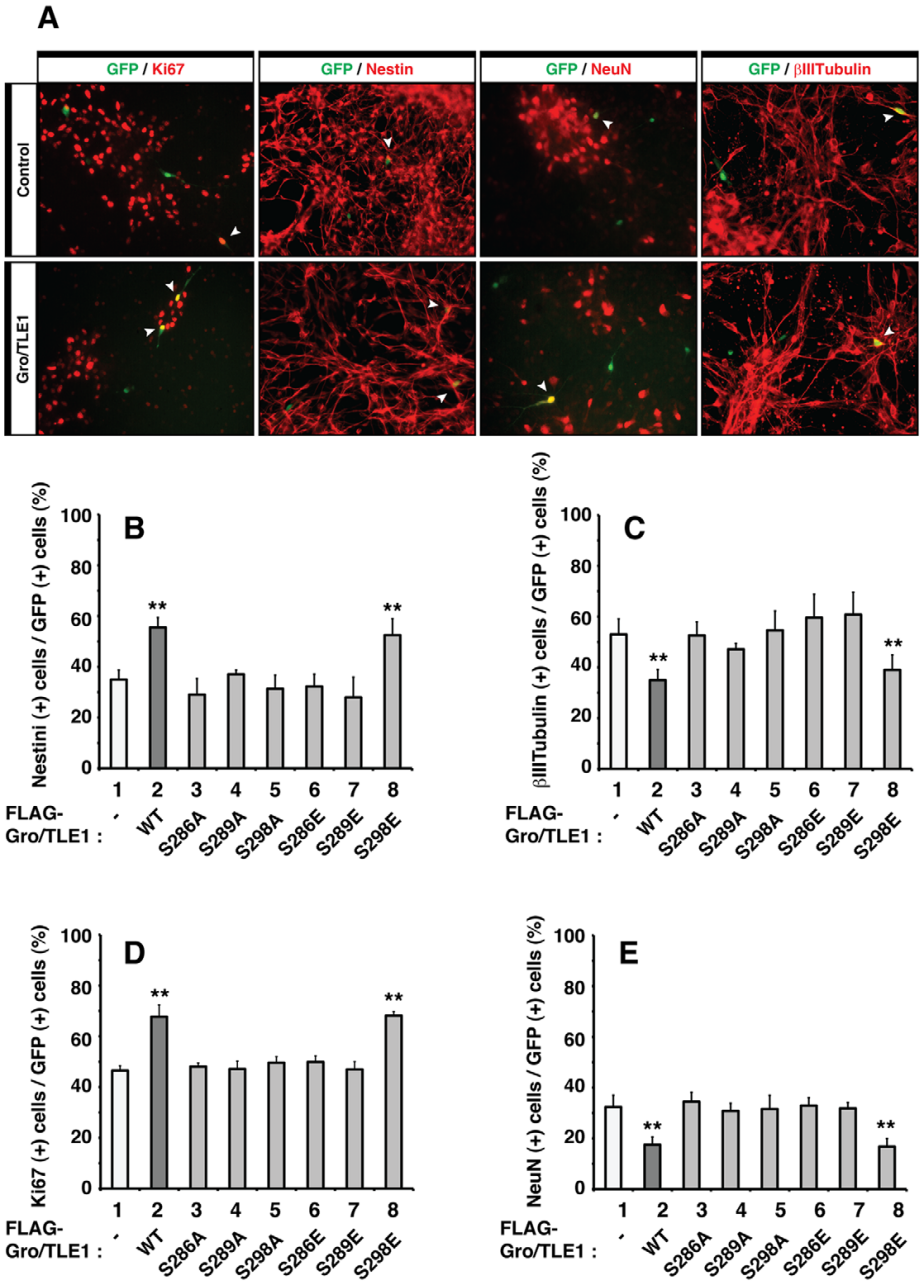
Effect of SP domain mutations on Gro/TLE1 ability to inhibit cortical neuron differentiation. (A) Primary cultures of E13.0 mouse embryonic cortical progenitor cells were transfected with plasmids encoding either GFP alone (Control; top row) or a combination of GFP and wild type Gro/TLE1 (Gro/TLE1; bottom row). After fixation, cells were subjected to double-labeling analysis of the expression of GFP (green) and either Ki67, nestin, NeuN, or type III β-tubulin (βIIItubulin) (red), as indicated. Arrowheads point to examples of double-labeled cells. (*B–E*) Quantitation of the percentage of GFP-positive cells that co-expressed nestin (B), type III β-tubulin (C), Ki67 (D), or NeuN (E) in the absence or presence of wild type or mutated forms of Gro/TLE1. The anti-neurogenic activity of Gro/TLE1 was impaired by the SP mutations S286A, S289A, and S298A. Results are shown as the mean±the standard deviation (>500 cells were counted in each case; n≥5; **, *P*<0.001 using the Student's *t* test).

Primary cultures of neural progenitor cells were established from E12–E13 mouse embryonic dorsal telencephalon [19], [20], [33]. These cortical progenitor cell cultures have been used extensively to investigate extrinsic and intrinsic regulators of cortical neuron differentiation [34], [35], [39], [40]. Wild type or mutated forms of Gro/TLE1 were expressed in cortical progenitor cells, together with enhanced GFP to visualize the transfected cells. Three days after transfection, immunofluorescence analysis was performed to determine the numbers of GFP-positive cells co-expressing markers of either proliferating undifferentiated neural progenitors or post-mitotic neurons (Fig. 7A). Under those conditions, less than 10% of the transfected cells normally corresponded to astrocytes at the time of analysis, and the number of cells undergoing programmed cell death was below 5% [20]. As previously shown [19], [20], exogenous expression of wild type Gro/TLE1 caused an increase in the number of cells co-expressing GFP and the mitotic neural progenitor markers nestin and Ki67, compared to control conditions (Fig. 7B and D, cf. bars 1 and 2). In parallel, wild type Gro/TLE1 caused a reduction in the number of GFP-positive cells exhibiting a neuronal morphology and expressing neuronal cell markers like type III β-tubulin and NeuN (Fig. 7C and E, cf. bars 1 and 2). Comparison of wild type and mutated forms of Gro/TLE1 showed that all those SP domain mutations that blocked cofactor-activated phosphorylation and weakened the strength of the association of Gro/TLE1 with chromatin also impaired the anti-neurogenic effect of Gro/TLE1 (Fig. 7B–E, cf. bars 2–7). In contrast, the S298E mutation, which had no effect on either cofactor-activated phosphorylation or affinity for chromatin, did not perturb the ability of Gro/TLE1 to inhibit neuronal differentiation (Fig. 7B–E, cf. bars 2 and 8). Taken together, these findings demonstrate a requirement of specific serine residues for Gro/TLE1 anti-neurogenic activity. Moreover, they provide evidence that phosphorylation changes induced by cofactor binding are essential to the ability of Gro/TLE1 to inhibit neuronal differentiation.

## Discussion

In this paper, we sought to determine whether the change in phosphorylation induced by transcriptional cofactor binding plays a positive or negative role in the ability of Gro/TLE1 to inhibit/delay the transition of cortical progenitor cells into neurons. Using specific point mutations in the SP domain of Gro/TLE1, we were able to uncouple cofactor interaction from cofactor-activated phosphorylation. Our studies showed that Gro/TLE1 anti-neurogenic function is disrupted by mutations that do not perturb interactions with DNA-binding partners but prevent cofactor-activated phosphorylation. This finding points to an essential role for cofactor-activated phosphorylation in Gro/TLE1 anti-neurogenic function.

### Role of SP Domain Ser-286 in Cofactor-Activated Phosphorylation

Through a combination of mass spectrometry and mutation analysis, we obtained evidence that Ser-286 of Gro/TLE1 (SS^286^ **S**PASTASS) is phosphorylated *in vivo*. This result is in agreement with *in vitro* studies in *Drosophila* showing the Ser-285 of Gro (position equivalent to Ser-286 of Gro/TLE1) is phosphorylated [29]. The identity of the kinase that might phosphorylate Ser-285/286 of Gro/TLE proteins remains to be determined. Computer-assisted prediction of possible phosphorylation sites (http://scansite.mit.edu) suggests glycogen synthase kinase 3 (GSK-3) as a possible kinase for Gro/TLE1 Ser-286. GSK-3 is normally constitutively active in cells [41]. This property would make GSK-3 a possible candidate for phosphorylation of target residues that are not always available but become exposed in regulated manners, as is likely the case for Gro/TLE residues that are phosphorylated in response to cofactor binding. GSK-3 has a loose substrate specificity, **S**/**T**-X-X-X-pS/pT, where a proline is often found near the phosphorylated site (highlighted in bold face type). Prior phosphorylation at adjacent residues is often, but not always, a requirement [41]. Across Gro/TLE family members, the sequence surrounding Ser-285/286 has a high density of phosphorylatable residues that could contribute to the susceptibility of Ser-285/286 to GSK-3-mediated phosphorylation. Our observation that Ser-289 of Gro/TLE1 is also required for cofactor-activated phosphorylation is consistent with that possibility, although we have not yet obtained evidence that Ser-289 is phosphorylated *in vivo*.

Regardless of the precise identity of the kinase involved in the phosphorylation of Ser-286, our present results demonstrate an important role for this residue in Gro/TLE1 cofactor-activated phosphorylation. Mutation of Ser-286 to either alanine or glutamic acid does not disrupt the ability of Gro/TLE1 to translocate to the nucleus and form functional transcription complexes with DNA-binding proteins, including Hes1. However, mutation of Ser-286 prevents cofactor-activated phosphorylation of Gro/TLE1. One interpretation of these results is that Ser-286 is not critical to the folding and protein-protein interaction ability of Gro/TLE1 but is a target of cofactor-activated phosphorylation. Alternatively, this residue might be phosphorylated independent of cofactor binding, with this phosphorylation needed for subsequent phosphorylation induced by cofactor interaction. We detected Ser-286 phosphorylation in HEK293 cells through mass spectrometric analysis of exogenous Gro/TLE1 without the need for co-transfection of DNA-binding partners such as Hes1. Although this finding might suggest that phosphorylation of Ser-286 is constitutive, it should be noted that HEK293 cells endogenously express Gro/TLE-binding proteins [36]. Thus, cofactor-activated phosphorylation might have occurred to a certain degree due to the presence of endogenous Gro/TLE-binding transcription factors. Taken together, our present studies provide evidence that Ser-286 is phosphorylated *in vivo* and is required for Gro/TLE1 phosphorylation in response to cofactor binding.

### Contribution of SP Domain Ser-289 and Ser-298 to Cofactor-Activated Phosphorylation

Our studies also identified Ser-289 and Ser-298 of Gro/TLE1 as residues important for cofactor-activated phosphorylation. Although evidence that these residues are phosphorylated *in vivo* is lacking, previous studies have shown that *Drosophila* Gro Ser-297, orthologous to Ser-298 of Gro/TLE1, is phosphorylated *in vivo* [29]. Moreover, evidence exists suggesting the phosphorylation of Gro Ser-287, which is analogous to Ser-289 of Gro/TLE1 [29].

We found that mutations of Ser-289 and Ser-298 have no deleterious effects on the folding, nuclear localization, and protein-protein interaction ability of Gro/TLE1. However, replacement of Ser-289 with either alanine or glutamic acid completely blocks Gro/TLE1 hyperphosphorylation induced by cofactor binding, as is the case with Ser-286 mutations. This finding suggests that Ser-289 might also be a target of cofactor-activated phosphorylation, if this residue were indeed phosphorylated *in vivo*. Alternatively, Ser-289 might be needed indirectly for cofactor-activated phosphorylation to occur, possibly at Ser-286.

Somewhat different results were obtained when the role of Ser-298 was examined. We observed that the S298A mutation causes a reduced, but still detectable, change in Gro/TLE1 phosphorylation in response to cofactor binding. In contrast, the S298E mutation does not perturb this process to a detectable extent. Taken together, these findings suggest that Ser-298 might be phosphorylated, but not as a direct target of cofactor-activated phosphorylation. Instead, phosphorylation of Ser-298 might be a contributing, but not essential, event to the latter. Optimal cofactor-activated phosphorylation might be achieved when Ser-298 is phosphorylated, a situation that could be mimicked by a serine-to-glutamic acid substitution. Alternatively, Ser-298 phosphorylation might be a parallel, additive event that could occur in response to cofactor binding but also independent of the latter. We found no evidence that the S298E mutant migrated more slowly than wild type Gro/TLE1 in the absence of exogenous cofactor. This situation suggests that this mutation does not cause a constitutive hyperphosphorylation, suggesting that phosphorylation of Ser-298 is not alone sufficient to activate a process like cofactor-activated phosphorylation.

Studies in *Drosophila* suggested that Gro Ser-297 is phosphorylated by *Drosophila* homeodomain interacting protein kinase 2 (DHIPK2), a nuclear Ser/Thr kinase that phosphorylates a variety of transcriptional regulators [42]. However, it remains to be determined whether or not HIPK2 phosphorylates Ser-297/298 of insect and vertebrate Gro/TLE proteins. This ambiguity derives from the fact that studies suggesting DHIPK2-mediated phosphorylation of Ser-297 of Gro were based on the use of a multiple point mutant in which Ser-297 was mutated together with Ser-194, Ser-196, Ser-285, and Ser-287 [29]. Moreover, Ser-297/298 is not followed by a proline residue, as is the case with most characterized HIPK2 substrates. This feature is consistent with the fact that the HIPK2 kinase domain is a p38MAPK-like domain [42]. In summary, the analysis of mutations of Ser-289 and Ser-298 show that these residues are also important for the ability of Gro/TLE1 to undergo cofactor-activated phosphorylation, although it remains to be determined whether or not they represent sites of *in vivo* phosphorylation.

### Impairment of Gro/TLE1 Cofactor-Activated Phosphorylation Is Correlated with a Weakened Ability to Associate with Chromatin

In addition to presenting evidence that Ser-286 is phosphorylated *in vivo*, our studies also showed that the mutations S286A and S286E do not perturb the nuclear translocation, interaction with DNA-binding proteins, and recruitment of Gro/TLE1 to chromatin. However, these mutations both impair cofactor activated phosphorylation and weaken the strength of Gro/TLE1 association with chromatin. It is worth mentioning that the findings that mutations of Ser-286 do not detectably alter the localization of Gro/TLE1 to chromatin but weaken the strength of its association with the latter are only apparently contradictory. The covalent cross-linking step that is part of the chromatin immunoprecipitation protocol used to determine association with specific promoters links the entire complex of Gro/TLE1 and its DNA-binding partners (e.g., Hes1) to chromatin. As a result, the association of the Gro/TLE1: Hes1 complex with DNA depends on the cross-linking to chromatin of the entire complex, including DNA-bound Hes1 and other components of the complex, and not just on the affinity of Gro/TLE1 for chromatin. This is not the case in subcellular fractionation assays that are performed in the absence of any cross-linking agent and thus can reveal changes in affinity for chromatin.

The finding that Ser-286 mutations weaken the chromatin association of Gro/TLE1 is consistent with the demonstration that pharmacological inhibition of protein kinase CK2-mediated phosphorylation, which occurs constitutively and is required for cofactor-activated phosphorylation, also weakens the association of Gro/TLE1 with subnuclear structures like chromatin and, possibly, the nuclear matrix (this study and [19], [27], [43]). Taken together, these observations suggest that Gro/TLE1 proteins carrying SP domain mutations that perturb cofactor-activated phosphorylation can become recruited to DNA but associate more weakly that wild type Gro/TLE1 with chromatin components.

### Cofactor-Activated Phosphorylation Is Required for Gro/TLE1 Anti-Neurogenic Activity

Prior to this study, the physiological significance of Gro/TLE phosphorylation in response to cofactor binding was not defined. A negative regulatory role for SP domain phosphorylation was suggested by the previous demonstration that phosphorylation of *Drosophila* Gro by DHIPK2 inhibits Gro-mediated transcriptional repression [29]. However, the mutant form of Gro used in those studies contained, in addition to mutations of Ser-285, Ser-287, and Ser-297, other serine-to-alanine substitutions at positions 194 and 196. The latter residues are part of a separate domain of Gro/TLE termed the protein kinase CK2/cell cycle-dependent kinase/nuclear localization sequence domain [44]. This domain was shown to also undergo phosphorylation [1], [19]. Additional evidence suggesting that phosphorylation of sites within the SP domain acts to down-regulate Gro/TLE function comes from separate studies showing that receptor tyrosine kinase-dependent MAPK pathways phosphorylate the Gro SP domain. This phosphorylation was mapped to Thr-308 (equivalent to Thr-312 in Gro/TLE1) and was shown to inhibit Gro-mediated transcriptional repression *in vivo* [45].

We were able to assess the role of cofactor-activated phosphorylation by taking advantage of the demonstration that the SP domain mutations characterized in this study block this hyperphosphorylation event but do not perturb the ability of Gro/TLE1 to functionally interact with DNA-binding proteins and become recruited to DNA. We found that all those mutations that disrupt or reduce cofactor activated phosphorylation, such as S286A/E, S289A/E, and S298A, also inhibit the anti-neurogenic activity of Gro/TLE1. In contrast, the S298E mutant, which undergoes an apparently normal cofactor-activated phosphorylation, has an intact anti-neurogenic ability. These results provide evidence that the change in Gro/TLE1 phosphorylation associated with cofactor binding is a positive mechanism that is required for the ability of Gro/TLE1 to inhibit neuronal differentiation.

Taken together, the present findings suggest that cofactor-activated phosphorylation is required for the anti-neurogenic activity of Gro/TLE1, and possibly for some of its other functions, by bringing changes in Gro/TLE1 phosphorylation that are correlated with the establishment of functional ‘repressosomes’ where Gro/TLE1 is in a complex with the appropriate transcription repression partners and forms stable interactions with transcriptionally relevant chromatin structures. In turn, this would result in optimal Gro/TLE-mediated transcription repression activity, possibly via chromatin remodelling mechanisms. This model might explain why disruption of cofactor-activated phosphorylation impairs the anti-neurogenic activity of Gro/TLE1, which involves regulation of endogenous promoter/enhancer elements, but does not detectably perturb Gro/TLE1-mediated transcriptional repression of exogenous reporter genes, which are unlikely to acquire a true chromatin-like structure in transfected cells. In the context of transiently transfected cells, Gro/TLE1 may be able to repress transcription via inhibition of the RNA polymerase complex, independent of its ability to modify chromatin structure. This partial activity might not be sufficient in the context of differentiating neural progenitor cells, where a combination of transcription repression activities, including chromatin remodeling mechanisms, is likely to underlie the *in vivo* functions of Gro/TLE1 [1].

The present, and previous [29], [45], findings suggest further that Gro/TLE activity may be controlled by antagonistic post-translational modifications that either promote (cofactor-activated phosphorylation) or inhibit (HIPK2 and/or MAPK-mediated phosphorylation) its function. It is possible that Gro/TLE proteins are activated by cofactor binding and the ensuing changes in phosphorylation, and that their activity persists until regulated phosphorylation mediated by HIPK2 and/or MAPK acts as a switch to stop Gro/TLE-mediated functions. This negative effect could be caused by weakening the interaction of Gro/TLE with chromatin and/or their transcriptional partners, or by changes in Gro/TLE stability and/or sub-nuclear distribution, ultimately resulting in weakened/blocked transcriptional repression. Future investigations will be aimed at testing these and other possibilities.

## Supporting Information

Figure S1 Analysis of the chromatin association of Gro/TLE1 by subcellular fractionation. Postnuclear supernatant and chromatin-enriched nuclear extracts from cells transfected with the indicated combinations of FLAG-tagged proteins were subjected to SDS-polyacrylamide gel electrophoresis (10% gel), followed by sequential Western blotting with antibodies against the FLAG epitope (a and c) or the p65 subunit of the NF-κB complex (b), as indicated. The nuclear association of Gro/TLE1 was weakened by the SP mutations S286A, S289A, and S298A. No changes were observed in the strength of the nuclear association of Hes1 and p65 (panels b and c). More slowly migrating forms (ie, hyperphosphorylated) of Gro/TLE1 were not resolved on a 10% SDS-polyacrylamide gel. A non-specific band is present in panel a, lane 2. Shown is a representative example of three separate experiments.
(0.15 MB PDF)

Figure S2 Chromatin immunoprecipitation experiments. AtT20 cells were transfected with Hes1 bearing no epitope tag and the indicated FLAG epitope-tagged Gro/TLE1 proteins, followed by chromatin immunoprecipitation assays using anti-FLAG (A–C) or control anti-GST (A) or anti-HA (B and C) antibodies, as shown. PCR amplification of each input chromatin (IN) and immunoprecipitated material using oligonucleotide primers flanking two canonical Hes1-binding sites located in the promoter region of mouse *Ascl1* yielded a 238 bp product only when the anti-FLAG antibody was used. Gro/TLE1 was specifically recruited to the *Ascl1* promoter and no significant differences in the ability of wild type and SP domain mutated Gro/TLE1 proteins to become recruited to the *Ascl1* promoter *in vivo* was detected, with some occasional variability observed across several experiments. (A and B) Lanes 5, 9, and 13 were empty. (C) Gro/TLE1 was not transfected in lanes 5–7. In all panels, lane 1 was loaded with molecular weight markers.
(0.23 MB PDF)

Supplemental Methods S1 (0.06 MB PDF)
