# Supplementary figures and images for: Cofactor-Activated Phosphorylation Is Required for Inhibition of Cortical Neuron Differentiation by Groucho/TLE1
Source: PLoS One. 2009 Dec 1;4(12):e8107. doi: 10.1371/journal.pone.0008107 (PMC2779591; doi:10.1371/journal.pone.0008107)

**Figure S1**

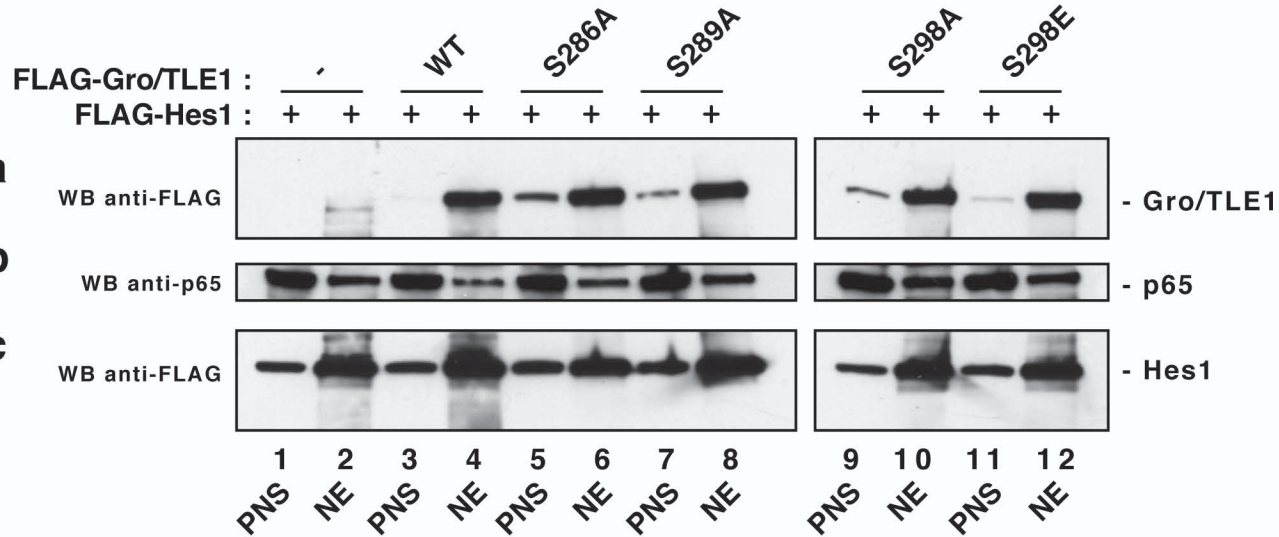

Supplement: Figure S1 — Analysis of the chromatin association of Gro/TLE1 by subcellular fractionation. Postnuclear supernatant and chromatin-enriched nuclear extracts from cells transfected with the indicated combinations of FLAG-tagged proteins were subjected to SDS-polyacrylamide gel electrophoresis (10% gel), followed by sequential Western blotting with antibodies against the FLAG epitope (a and c) or the p65 subunit of the NF-κB complex (b), as indicated. The nuclear association of Gro/TLE1 was weakened by the SP mutations S286A, S289A, and S298A. No changes were observed in the strength of the nuclear association of Hes1 and p65 (panels b and c). More slowly migrating forms (ie, hyperphosphorylated) of Gro/TLE1 were not resolved on a 10% SDS-polyacrylamide gel. A non-specific band is present in panel a, lane 2. Shown is a representative example of three separate experiments. (0.15 MB PDF) [file pone.0008107.s001.pdf]

**Figure S2**

**A**

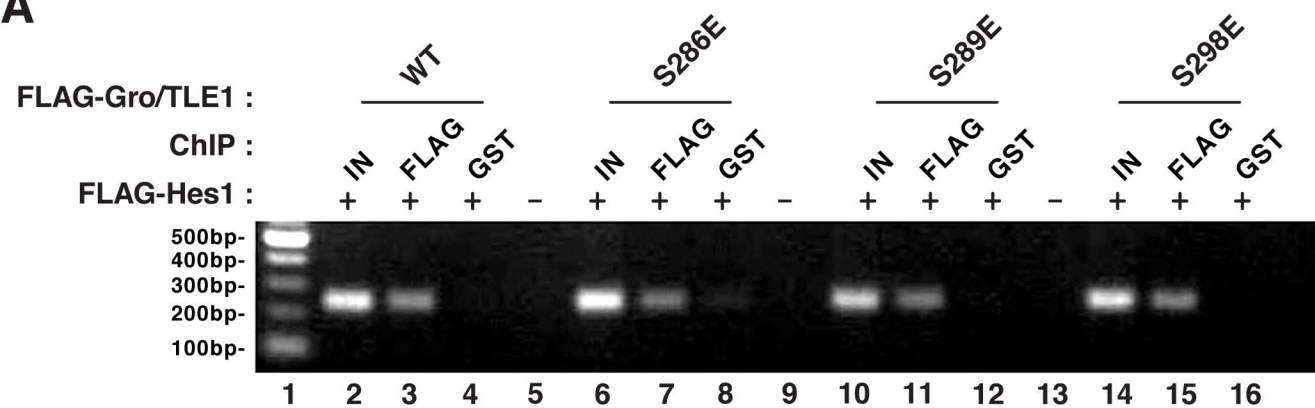

**B**

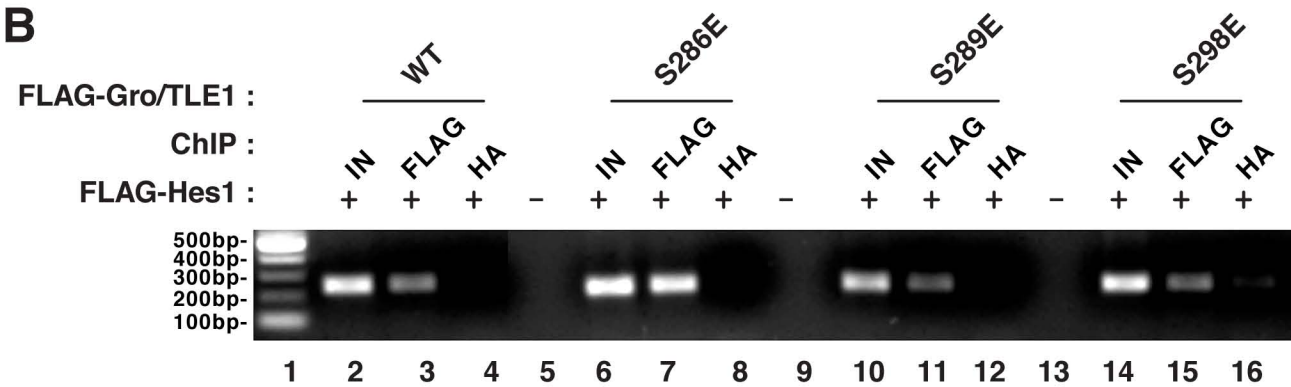

**C**

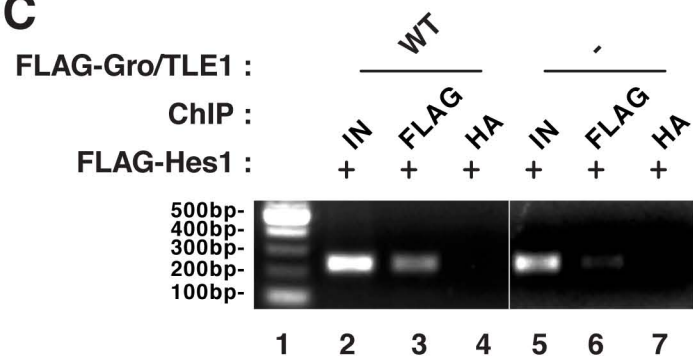

Supplement: Figure S2 — Chromatin immunoprecipitation experiments. AtT20 cells were transfected with Hes1 bearing no epitope tag and the indicated FLAG epitope-tagged Gro/TLE1 proteins, followed by chromatin immunoprecipitation assays using anti-FLAG (A–C) or control anti-GST (A) or anti-HA (B and C) antibodies, as shown. PCR amplification of each input chromatin (IN) and immunoprecipitated material using oligonucleotide primers flanking two canonical Hes1-binding sites located in the promoter region of mouse Ascl1 yielded a 238 bp product only when the anti-FLAG antibody was used. Gro/TLE1 was specifically recruited to the Ascl1 promoter and no significant differences in the ability of wild type and SP domain mutated Gro/TLE1 proteins to become recruited to the Ascl1 promoter in vivo was detected, with some occasional variability observed across several experiments. (A and B) Lanes 5, 9, and 13 were empty. (C) Gro/TLE1 was not transfected in lanes 5–7. In all panels, lane 1 was loaded with molecular weight markers. (0.23 MB PDF) [file pone.0008107.s002.pdf]
