# Supplementary material for: Cofactor-Activated Phosphorylation Is Required for Inhibition of Cortical Neuron Differentiation by Groucho/TLE1
Source: PLoS One. 2009 Dec 1;4(12):e8107. doi: 10.1371/journal.pone.0008107 (PMC2779591; doi:10.1371/journal.pone.0008107)
Supplement: Supplemental Methods S1 — (0.06 MB PDF) [file pone.0008107.s003.pdf]

Supplemental Methods S1

Chromatin immunoprecipitation

Chromatin immunoprecipitation experiments were performed using the Magna ChIP G chromatin immunoprecipitation kit (Millipore, Billerica, CA). All steps were performed according to the manufacturer's instructions. Mouse AtT20 pituitary cells (26) were transfected with plasmids encoding FLAG epitope-tagged wild type or mutated Gro/TLE1 proteins using the FuGENE HD reagent as instructed by the manufacturer (Roche Applied Science, Indianapolis, IN). The DNA plasmid pRc/CMV-Hes1 was used to co-express a form of Hes1 with no epitope tags. Chromatin of transfected AtT20 cells was cross-linked for 10 min using formaldehyde, sonicated to yield 200-600 bp fragments, and subjected to immunoprecipitation with either anti-FLAG (Sigma), anti-HA (Covance), or anti-GST (Santa Cruz Biotechnologies) antibodies. Chromatin:antibody complexes were harvested using protein G magnetic beads and a magnetic separator, washed extensively, incubated at 62 ^{o}C in the presence of "ChIP Elution Buffer" containing 0.1 mg/ml proteinase K (supplied by the manufacturer) to reverse the cross-linking and recover the DNA. Immunoprecipitated DNA was purified using spin columns and reagents supplied with the Magna ChIP G kit and analyzed for the mouse *Ascl1* promoter sequence by PCR using oligonucleotide primers whose respective 5' ends were located 439 bp (primer *Ascl1p-F*) and 201 bp (primer *Ascl1p-R*) upstream from the starting ATG codon, resulting in a product of 238 bp. The amplified region contains two tandem copies (<u>CACGCG</u>AGCGC<u>CACGCG</u>) of the N-box, a canonical Hes binding site (10, 17, 26, 32). These sequences correspond to the two previously described tandem Hes1-binding sites in the proximal region of the rat *Ascl1* gene (26). The sequence of the oligonucleotide primers was as follows:

Ascl1p-F, 5’-TCAAGCCCAGGCTGGAGCAAG-3’; *Ascl1p-R*, 5’-GGCGATCGTCTTCCCTCTGCG-3’.
